# Radiographic Features of Mandibular Second Molars with Eruption Disturbances: A Retrospective Study

**DOI:** 10.3390/jcm12082798

**Published:** 2023-04-10

**Authors:** Qianyun Kuang, Hong Zhou, Huiyi Hong, Donger Lin, Meng You, Wenli Lai, Hu Long

**Affiliations:** 1 State Key Laboratory of Oral Diseases, National Clinical Research Center for Oral Diseases, Department of Orthodontics, West China Hospital of Stomatology, Sichuan University, Chengdu 610041, China; 2 State Key Laboratory of Oral Diseases, National Clinical Research Center for Oral Diseases, Department of Oral Radiology, West China Hospital of Stomatology, Sichuan University, Chengdu 610041, China

**Keywords:** mandibular second molar, eruption disturbances, retention, impaction, radiograph

## Abstract

We aimed to establish the characteristics and potential etiological risk factors of eruption disturbances in mandibular second molars (MM2). We retrospectively enrolled patients with eruption disturbances in MM2. A total of 143 MM2 with eruption disturbances from 112 patients (mean age 17.45 ± 6.35) were included in this study. Panoramic radiographs were employed to determine the risk factor, angulation type, impaction depth, tooth development stage, and associated pathology. The novel classification method of MM2 was based on impaction depth and angulation. Of 143 MM2, 137 and 6 were diagnosed with impaction and retention, respectively. Insufficient space was the most frequent risk factor for eruption disturbances. There were no significant differences between retention and impaction regarding sex, age, or side. The most frequent impaction type was Type I. The most frequent angulation of impacted MM2 was mesioangular. Impacted MM2 with shallower depth were more frequently associated with the presence of first molar undercut. Impaction types did not differ according to age, side, development stage, or distance from the MM1 distal surface to the anterior border of the ramus. Dentigerous cysts were associated with earlier MM2 development stages and greater MM2 depth. In conclusion, MM2 impaction types differed according to the risk factor, angulation type, MM1 undercut, and presence of cysts. Early MM2 development stage and greater MM2 depth were risk factors for MM2 eruption disturbances with cysts.

## 1. Introduction

Eruption disturbances of mandibular second molars (MM2), often caused by the ectopic position of tooth germs, obstacles in the eruption path, and failures in the eruption mechanism [1,2], is a rare condition occurring in approximately 0.65–3% of the general population [3,4,5]. However, recently, the reported incidence has been increasing [6,7,8,9], which could be attributed to more frequent radiographic examinations. Impacted MM2 are the most common eruption disturbances among mandibular molars, apart from impacted mandibular third molars (MM3). Both systemic and local factors contribute to this eruption disorder [5], including the distance between the mandibular first molar (MM1) and MM2 [10], early eruption of MM3 [11], crowding in the posterior sector [12], and abnormal inclination of the MM2 bud [13].

**Figure 1:**
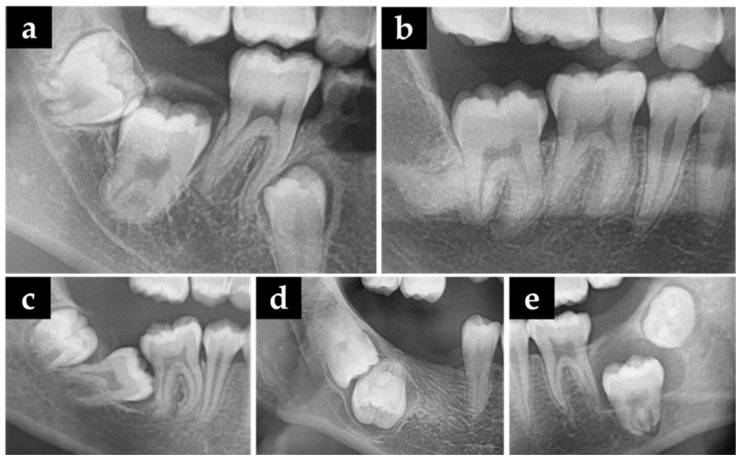
Panoramic radiographs revealed risk factors for eruption disturbances. (**a**) Primary retention; (**b**) Secondary retention; (**c**) Impaction due to insufficient posterior space; (**d**) Impaction due to ectopic eruption path; (**e**) Impaction due to cyst of jaw.

Eruption disturbances result in retention or impaction of the tooth concerned (Figure 1). Retention is defined as an eruption disturbance without a physical obstacle in the eruption path or an abnormal position and is classified as primary and secondary retention based on the suspension of eruption before and after emergence, respectively [14]. Secondary retention is mainly caused by ankylosis [15]. Impaction is defined as the failure of tooth eruption due to a clinically or radiologically detectable physical barrier in the eruption path or an abnormal direction of tooth eruption [14,16].

Eruption disturbances of the mandibular molars cause a significant clinical effect because permanent molars are crucial for the development of dentition and coordination of facial growth. Moreover, eruption disturbances can cause a variety of diseases, such as caries in adjacent teeth, pericoronitis, temporomandibular joint symptoms, cysts, and facial infections [17]. Previous studies have focused on the factors associated with MM3 impaction [18,19]; however, studies on eruption disturbances of MM2 are limited and are mainly treatment-oriented. Moreover, to the best of our knowledge, few studies have explored the impaction characteristics of MM2. Therefore, the objective of this study was to determine the eruption disturbances characteristics of MM2 and to identify potential etiological factors associated with MM2 eruption failure.

## 2. Materials and Methods

We conducted a retrospective study using all panoramic radiograph data from 2016 to 2021 at West China Dental Hospital, screening all cases with MM2 eruption disturbances.

### 2.1. Study Population

This retrospective clinical study involved the radiographic histories of 112 patients (67 males and 45 females) with MM2 eruption failure selected from those who had panoramic radiographs taken at the West China Hospital of Stomatology, Sichuan University between 2016 and 2021; all the patients’ ethnicity were Han Chinese. A total of 143 MM2 with retention or impaction were found among the 112 patients. The overall age of the included patients was 17.45 ± 6.35 years (17.73 ± 6.80 for men, 15.15 ± 5.14 for women).

The inclusion criteria were as follows: (1) bilateral or unilateral MM2 eruption disturbances and (2) availability of high-quality panoramic radiographs. The exclusion criteria included those who were undergoing orthodontic treatment at the time when radiographs were taken. These patients were excluded since the initial positions of the second molars with eruption disturbances may be changed due to orthodontic treatments. Otherwise, the initial spatial positions and risk factors of the second molars with eruption disturbances may be misdiagnosed.

### 2.2. Study Variables

All cases of eruption disturbances were screened by an experienced radiologist and then reviewed by an experienced orthodontist. Usually, second molars with eruption disturbances may be associated with several causes, e.g., cysts, ectopic eruption path, and insufficient space. In these clinical scenarios, the primary cause of the eruption disturbances is the most apical or the first obstacle that impedes the eruption of second molars. As second molars erupt from the apical level to the occlusal level, the most apical or first obstacles that interfere with their eruptions were considered the primary causes. For example, an impacted second molar is associated with a cyst and an overlying third molar. The cyst is considered as the first obstacle that interferes with the eruption of the second molar, and the overlying third molar is secondary to the non-eruption of the second molar. Thus, in this situation, the cyst is considered the primary cause of the eruption disturbances. The descriptive variables of interest included age, sex, unilateral and bilateral eruption disturbances, side of eruption disturbances, risk factor, undercut, cyst, posterior space of mandibular, tooth angulation, tooth development level, and classification of the depth of the impacted tooth.

Age was calculated as the date of the first captured MM2 eruption disturbances. Eruption disturbances of MM2 were classified as retention and impaction according to the criteria of Raghoebar et al. [14], where retention was classified as primary and secondary; impaction was classified as insufficient space, ectopic eruption path, and cyst according to the pathogenesis. Tooth angulation was categorized as horizontal, mesioangular, vertical, distoangular, or other. The level of MM2 development is classified into 11 stages, according to Haavikko [20]. We designed a modified classification of the depth level of MM3 impaction from the Pell and Gregory classification, which is more suitable for evaluating MM2 impaction into three classifications.

For the correlation between MM2 and cysts, cysts were the dependent variable. We excluded all cases with a diagnosis of retention since there was no association between cysts and retention, as well as performed a prediction of the correlation between impacted MM2 and cysts. Variables included age, sex, impaction depth, tooth angulation, stage of MM2 development, posterior space of mandibular, and undercut.

### 2.3. Image Analysis

All information on images was obtained through panoramic radiography. Each radiograph was interpreted by two experienced specialists. Measurements were repeated by the same researchers for the reliability of data.

#### 2.3.1. Radiological Differential Diagnosis of Eruption Disturbances

(1) *Primary retention* is represented on radiographs as the cessation of the eruption of a normally positioned and developed tooth before gingival emergence, with no identifiable physical barrier during the eruption.
(2) *Secondary retention* is displayed on radiographs as the cessation of the eruption of a normally positioned and developed tooth after gingival emergence, without a recognizable physical barrier in the eruption path, and with a lower vertical alveolar height than the rest of the tooth.
(3) *Impaction* is the arrest of the eruption of a tooth due to a detectable physical barrier in the eruption path or due to an abnormal position of the tooth.

#### 2.3.2. Data Collection

##### Patient Demographics and Baseline Information

(1) Risk factor: the etiology of MM2 eruption disturbances was classified as primary retention, secondary retention, and impaction. The risk factor of impaction included insufficient space, ectopic eruption path, and cyst of the jaw; (2) Unilateral or bilateral eruption failure: eruption disturbances occurring on both sides simultaneously or separately; (3) Side of MM2 eruption disturbances: whether unilateral eruption disturbances occurred on the left or right side in patients.

##### Measurements

Since the majority of the mandibular second molars were classified as impaction, patients with primary or secondary retention were excluded for further measurements and quantitative analyses. Thus, the following measurements and quantitative analyses were for impaction only.

**Figure 2:**
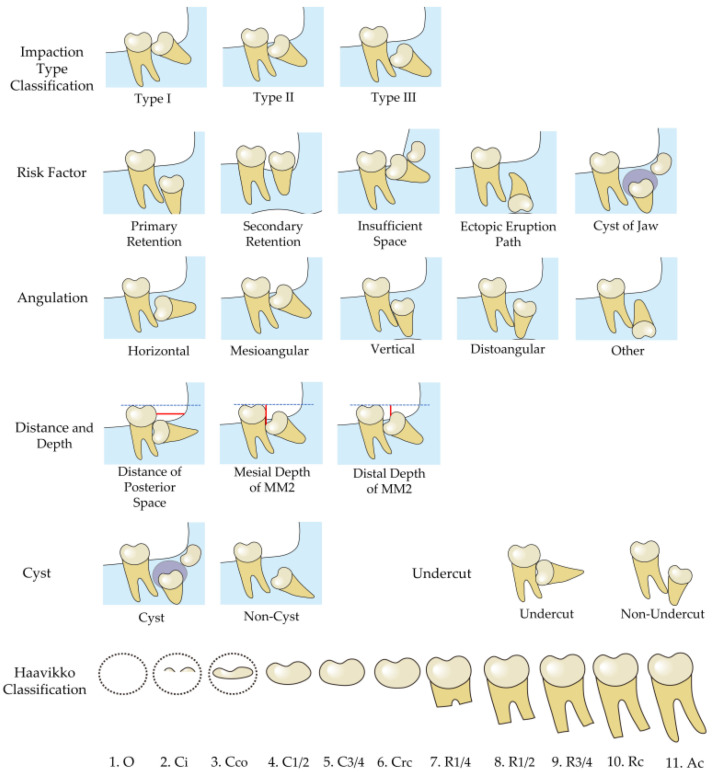
Data collection of MM2 impaction. Dash blue lines indicate the occlusal plane; red lines indicate distance or depth.

(1) Impaction type: Since the Pell and Gregory classification is only applicable to MM3, there is no suitable classification for eruption disturbances of MM2. Hence, we designed a modified classification according to the relationship between the depth and angle of MM2 relative to the adjacent MM1, as follows: (a) Type I: the most occlusal point of the MM2 crown lies at the occlusal half of the adjacent MM1 crown; (b) Type II: the most occlusal point of the MM2 crown lies at the gingival half of the adjacent MM1 crown; and (c) Type III: the most occlusal point of the MM2 crown lies apical to the cementoenamel junction of the adjacent MM1; (2) Depth of MM2 (in mm): distance between the occlusal plane, and the mesial or distal cusps of MM2 with eruption disturbances. The occlusal plane was the line passing through the buccal cusp of the mandibular first premolar and the buccal cusps of the mandibular first molar [21]; (3) Angulation type: angulation of the MM2 with eruption disturbances relative to the vertically erupted adjacent tooth, which was categorized as horizontal, mesioangular, vertical, distoangular, or other; (4) Evaluation of dental age: the Haavikko classification of dental developmental stages was used to classify the enrolled MM2 and MM3 into 11 stages according to the development of the crowns and roots [20]; (5) Cyst: the presence of cysts was evaluated; (6) Posterior mandibular space: distance from the most distal surface of MM1 to the anterior edge of ramus projected onto the occlusal plane; (7) Undercut: whether MM2 was located beneath the distal undercut of MM1 (Figure 2).

### 2.4. Statistical Analysis

The frequencies of risk factors, unilaterality or bilaterality, left or right side, different types of impaction, angulations of MM2, cyst, presence of undercut, and Haavikko staging were calculated. The associations between the type of MM2 impaction and other categorical variables (i.e., sex, impaction side, number of patients with unilateral and bilateral eruption disturbances, MM2 angulation, cyst, presence of undercut, risk factor, and Haavikko staging) were analyzed using chi-square tests. The differences in continuous variables (angle of molar inclination, impaction depth, distance from the distal surface of MM1 to the anterior border of the ramus) among impaction types were compared using one-way analysis of variance. The difference between the MM2 and MM3 Haavikko staging was compared using the Wilcoxon signed-rank test. The correlation between cysts associated with MM2 and other potential variables was analyzed using binary logistic regression.

Data were analyzed using SPSS (version 26.0; IBM SPSS Statistic, Armonk, NY, USA) and GraphPad Prism (version 8.02; GraphPad Software, San Diego, CA, USA). Statistical significance was set at *p* < 0.05. The normality test was performed through both the Shapiro–Wilk test and Kolmogorov–Smirnov test. The inter- and intra-examiner reliability was analyzed with the kappa reliability test and Student’s paired *t*-test.

## 3. Results

**Figure 3:**
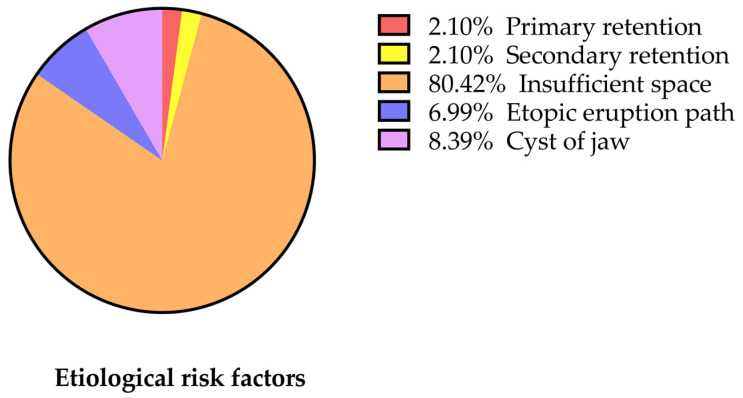
Etiological risk factors associated with the 143 eruption disturbances of MM2.

A total of 112 patients (67 males and 45 females) with a mean age of 17.45 ± 6.35 years were included in the study, with the majority of participants (81, 72.32%) having unilateral MM2 eruption disturbances. Among them, 46 (56.79%) and 35 (43.21%) patients had MM2 eruption disturbances on the left and right sides, respectively (Table 1). In total, 143 mandibular second molars with eruption disturbances were detected, with the majority of them being impaction (*n* = 137). Moreover, few of them were due to primary retention (*n* = 3) and secondary retention (*n* = 3) (Figure 3) (Table 2). Thus, since the majority of patients had impaction, our further analysis only included teeth with impaction.

The kappa values of inter- and intra-reliability in etiological risk factors were 0.92 and 0.90; type classification were 0.92 and 0.93; angulation classification were 0.95 and 0.91; Haavikko stage were 0.81 and 0.83; and undercut were 0.93 and 0.82. The above kappa values show almost perfect agreement in inter- and intra-examiner.

The *p*-values of inter- and intra-reliability in the mesial depth of MM2 were 0.76 and 0.73 (*p* > 0.05); the distal depth of MM2 were 0.59 and 0.50 (*p* > 0.05); the posterior distance of mandibular were 0.96 and 0.92 (*p* > 0.05). Both the Shapiro–Wilk and Kolmogorov–Smirnov normality tests indicated that all continuous data conformed to normal distributions (all *p* > 0.1).

Among all patients, 137 MM2 were impacted; 63 (45.99%), 38 (27.73%), and 36 (26.28%) were classified as Types I, II, and III, respectively (Table 3). Cysts were observed in 11 patients (Table 3).

All 137 impacted MM2 were classified between stages 8 and 11 using Haavikko staging, with a median stage of 10. Furthermore, 131 MM3 were staged between stages 1 and 11, with a median of 6.

**Figure 4:**
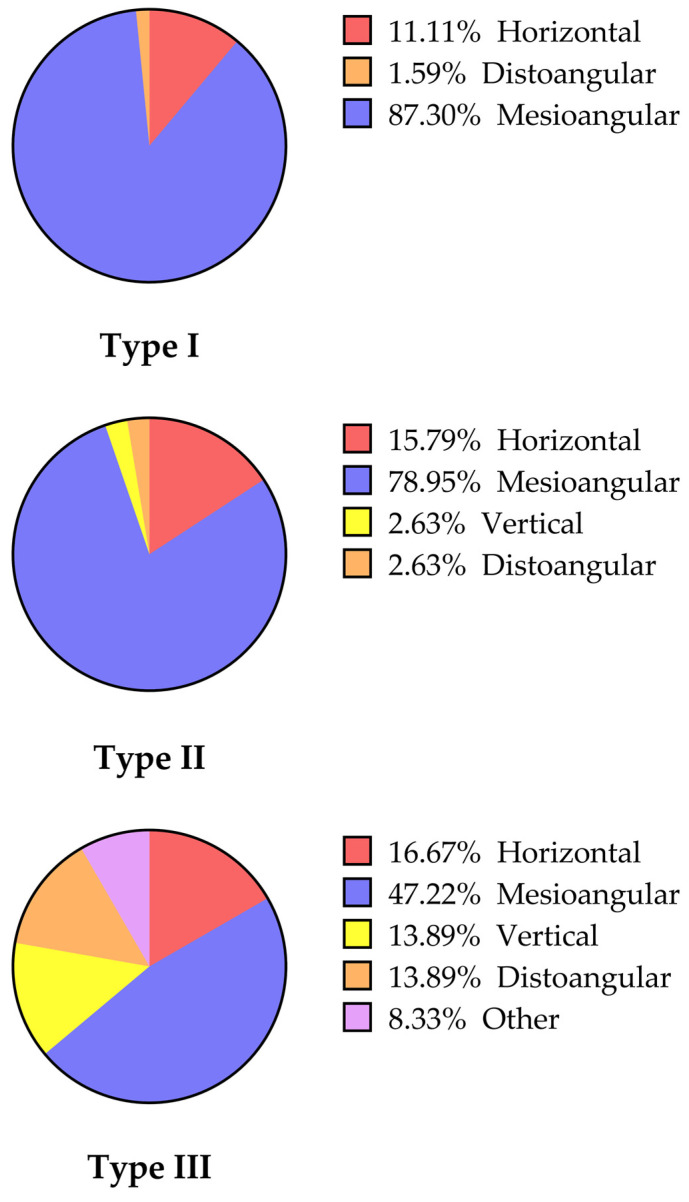
Impaction types of the 137 impacted MM2.

**Figure 5:**
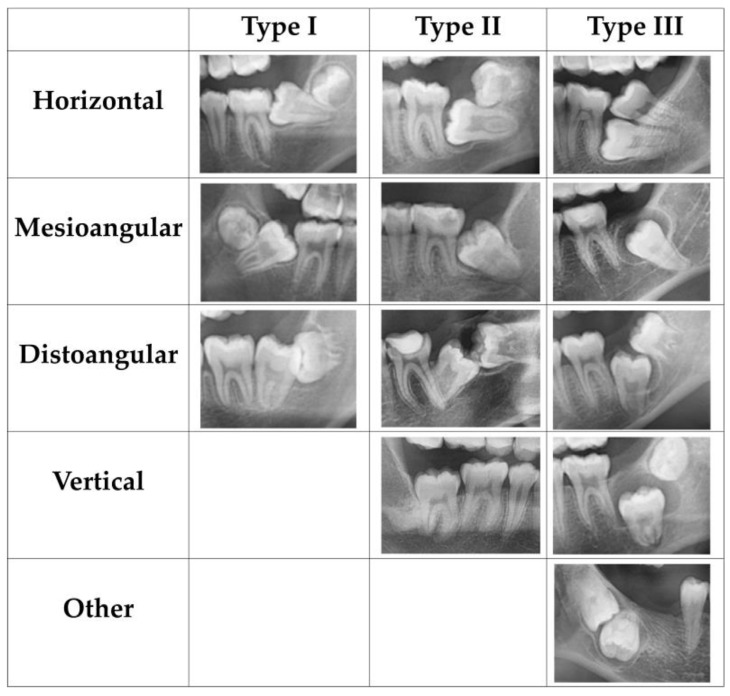
Panoramic radiographs with different angulations of MM2 between the three types.

Among the impacted MM2, 19 (13.87%), 102 (74.45%), 6 (4.38%), and 7 (5.11%) were classified as horizontal, mesioangular, vertical, and distoangular impactions, respectively, and 3 (2.19%) teeth were categorized as other angulations; the angle of impacted MM2 ranged from −29.1° to +166.31° (Table 3) (Figure 4 and Figure 5).

Significant differences in angulation type (*p* < 0.001), impaction depth (*p* < 0.001), risk factor of impaction (*p* < 0.001), presence of cyst (*p* < 0.001), and presence of undercut (*p* < 0.001) were found among the three impaction types (Table 3). The depth of both the mesial and distal cusps of MM2 with respect to the occlusal plane was the least in Type I, followed by Type II, and the greatest in Type III (*p* < 0.001). The incidence of cysts was the lowest in Type I, second in Type II, and the highest in Type III (*p* < 0.001), whereas MM2 occurred more frequently beneath the distal undercut of MM1 in Type I than in Types II and III (*p* < 0.001). In terms of angulation type, the distribution was significantly different among Types I, II, and III, with mesioangular impaction being the most prevalent (*p* < 0.001). However, no significant difference was observed in the number of patients with unilateral and bilateral retention/impaction (*p* > 0.9999), sex (*p* = 0.081), or impaction side (*p* = 0.311). In addition, there was no significant difference in the Haavikko stage of MM2 (*p* = 0.061) and the distance from the distal surface of MM1 to the anterior border of the ramus (*p* = 0.226) among the three impaction types (Table 1 and Table 3).

The results of the binary regression analysis of cysts around the impacted MM2 with other variables are presented in Table 4. Specifically, in the relationship between the impaction depth of MM2 and the concurrent occurrence of cysts, MM2 covered by bone were more likely to be accompanied by cysts than MM2 covered by mucosa (odds ratio [OR], 51.92; *p* < 0.001). Moreover, MM2 development was significantly associated with the occurrence of cysts: compared with patients with Stage 11 development, those with Stages 8, 9, and 10 were 229.97, 113.47, and 42.30 times more likely to have cysts (OR, 229.97; *p* < 0.01, OR, 113.47; *p* < 0.05, and OR, 42.30; *p* < 0.05, respectively) (Table 4).

## 4. Discussion

In this retrospective study, we found that impaction caused the majority of MM2 with eruption disturbances, whereas retention was the minority. Further evaluation of the risk factor of impacted MM revealed that most Type I or Type II MM2 were due to insufficient space, whereas the risk factors of Type III MM2 varied, including insufficient space, ectopic eruption path, and cyst. The dominant angulation of MM2 in Type I and II impaction was mesioangular, while there were various angulations of Type II. In addition, the odds of cyst development were associated with an increased depth of MM2 impaction and an earlier stage of MM2 development. Moreover, a cyst was not associated with sex, age, presence of undercut, narrower posterior space, or angulation.

The limitations of the study include that the source of the studied cases was a radiology database, in which not all patients with a diagnosis of MM2 eruption disturbances had the chief complaint of unerupted MM2, and not all patients had been treated for MM2. Therefore, we were unable to obtain follow-up radiographs and clinical examinations of all patients. Hence, only radiographs from a limited period could be evaluated.

In addition, selective bias may have occurred because the inclusion source of this study was not the population census but patients who spontaneously came to our hospital for radiographic examinations. Therefore, we reviewed all panoramic radiographs taken in our hospital during this period to maximize the sample size, and two experienced clinicians repeated the screening and measurements, aiming to minimize the impact and potential bias caused by study limitations.

CBCT three-dimensional (3D) imaging has been broadly available in the profession for the past two decades. CBCT has the capacity of providing information on several aspects and views for the examination of 3D integrity of the buccolingual aspects of the teeth, as well as cuts in various planes through the individual tooth. The question might arise regarding whether 3D images are better suited for research than the panoramic radiograph. However, the panoramic radiograph is still a powerful diagnostic tool and is the most popular radiograph taken from most patients as the first step in the diagnostic procedure. As such, it should be exploited as the means of deciding if a CBCT is necessary for an individual patient before exposing the patient to a greater dose of radiation than may be justified.

Several potential risk factors can be observed when analyzing a panoramic radiograph of MM2 eruption disturbances. In general, the primary risk factor of the eruption disturbances is the major or the first obstacle that impedes the eruption of second molars. As second molars erupt from the apical level to the occlusal level, thus the most apical or first obstacles that interfere with their eruptions were considered as the primary causes. Of these, the presence of MM3 could easily be mistaken as the primary risk factor for MM2 eruption disturbances. MM3 was often observed overlaying above MM2 in the panoramic radiograph. In effect, due to the eruption time of MM2 should be earlier than MM3, the primary risk factor of failure eruption of MM2 should be other factors, not caused by MM3 resistance.

Tooth eruption is determined by genetic and external factors, as well as the local environment. However, the mechanism of tooth eruption is unclear. Previous studies on tooth eruption [22,23,24] have shown that tooth eruption occurs due to the collaboration of different eruptive mechanisms. Some researchers believe that there is an association between tooth eruption and root extension; therefore, the interrelation between the periodontium and pulp influences tooth eruption. Similarly, the follicle that surrounds the crown influences tooth eruption. The failure of any of these mechanisms may lead to retention or impaction of the tooth.

MM2 eruption disturbances might interfere with normal masticatory function, resulting in occlusal instability and temporomandibular joint problems. In recent years, the incidence of MM2 eruption failure has been gradually increasing [6,7,8,25,26,27]; however, the related studies to date are mainly treatment-oriented, and few have focused on the etiology and pathological features of impacted MM2. Hence, our study, based on panoramic radiographs, aimed to reveal impaction-related characteristics of MM2 and to investigate potential factors affecting impaction severity. Additionally, we developed a precise method of classification, which provides clinicians with a comprehensive understanding of the characteristics and risk factors of MM2 impaction and a clear guideline for appropriate diagnosis and treatment planning, thereby preventing or reducing the sequalae caused by abnormal MM2.

Previous studies have established the prevalence of MM2 eruption disturbances (0.65–1%) in the Chinese population [6,8]. However, the actual prevalence of MM2 impaction may be underestimated since asymptomatic MM2 impaction is frequently overlooked by patients and during dental treatment without routine radiography. Shapira indicated a higher prevalence of MM2 eruption disturbances in Chinese Americans than in Israeli populations, and other studies indicated that MM2 impaction is more common in Chinese than in Caucasian populations, which is probably due to the greater tooth size in Chinese patients [28,29].

MM2 usually erupts between the ages of 10 and 13 years, whereas the mean age of patients in this study at the first panoramic examination was 17.45 ± 6.35 years. Particularly, the mean age of patients classified as having Type III impaction was the most advanced (18.61 ± 7.28 years) compared to the two other impaction types, which is probably due to the greater difficulty in detecting deeper tooth impaction by patients themselves. In terms of the proportion of the different sexes and impaction sides, previous studies have shown both similar and opposite results to our study, which may be related to different sample sizes and races in these studies [6,8,12,30].

The Pell–Gregory classification is commonly used to predict the difficulty in extracting impacted MM3. In the present study, a modified classification system of impaction type was designed for impacted MM2 based on the impaction depth relative to the adjacent MM1, as measured on panoramic radiographs, which can be used to better judge the difficulty in treatment of MM2 eruption disturbances. Moreover, the impaction type was associated with the retromolar space, undercuts, and presence of cysts, which were significantly different.

In all three impaction types, mesioangular impaction was the most prevalent angulation pattern, followed by horizontal and distoangular impactions. Notably, vertical and other angulations (fully buccal and lingual inclination that cannot be distinguished on panoramic radiographs) were only observed in Type III impactions, indicating that more directional impaction emerged in deeper impaction. Failure of MM2 eruption toward the occlusal plane may be due to the ectopic development of the tooth germ in other angulations, which causes it to be stuck in the alveolar bone.

Among the various risk factors of MM2 impaction, insufficient space was the most common risk factor for all three impaction types. Insufficient space in the mandible may be due to hereditary causes or may be caused by differences between mandibular growth and dental development. Compared to impactions caused by other risk factors, the mesiodistal width of the retromolar space was significantly smaller than that of the MM2 in cases with impactions directly caused by insufficient space, which is consistent with the findings of Padwa et al. [31]. Similar to angulation patterns, risk factor was also more diverse in Type III than in the other two types, including insufficient space, ectopic eruption path of MM3, and cysts, which suggest that deeper impactions tend to have more causes.

Kjær [23] concluded that primary retention might be caused by defects in the dental follicle; in particular, the epithelium of the dental follicle could be inefficient and incapable of initiating resorption of the overlying hard tissue. Failure of continued eruption of a single tooth after the clinical eruption is designated secondary retention. Trauma or acquired disturbances of the periodontal membrane can cause inflammation of the periodontal membrane where the root-close periodontium is, resulting in secondary retention; furthermore, blood and lymph exuding from the periodontal membrane may exert pressure that causes the breakdown of the normal periodontal structures and the deposition of hard tissue, leading to ankylosis [23].

It should be noted that the influence of MM3 on MM2 impaction remains controversial. In our study, 90.2% of the 143 MM2 with eruption disturbances had adjacent MM3, which is similar to the 85–100% reported in a previous study [32]. Despite the high prevalence of MM3, the relationship between the existence of MM3 and the impaction of MM2 has varied across previous studies; nevertheless, the common conclusion is that the existence of MM3 is not a risk factor for MM2 impaction [13,31,32].

Based on our findings, premature development of MM3 (Havvikko stages of MM2 and MM3 within two stages of each other) and abnormal eruption path of MM3 could lead to competition for eruption space between MM2 and MM3. There was one case of unilateral kissing molars in our study. Some researchers link kissing molars to hereditary mucopolysaccharidoses, whereas others believe it is a solitary radiological finding [33,34]; however, most researchers believe that kissing molars occur due to the expansion of a cyst that causes resorption of the mesial alveolar wall of the MM3, resulting in the inclination of MM3 towards the MM2.

Whether the abnormal MM3 is a result or the cause of the impaction remains controversial and requires further verification. Regardless of whether the MM3 is associated with MM2 impaction, early extraction of MM3 has been confirmed to increase the likelihood of spontaneous eruption of MM2; therefore, it is recommended that MM3 should be extracted before treating MM2 impaction.

A study by Ghougassian et al. showed that the developmental stage of the tooth was correlated with the retromolar space, with a 5-mm increase in the retromolar space corresponding to a 1.8 Nolla stage of MM3 development [35]. Hence, MM3 development might also cause the impaction of MM2 by occupying the mesiodistal width of the retromolar space, thus leading to insufficient space for MM2 eruption. Therefore, it is important to monitor MM3 development when treating MM2 eruption disturbances [36].

Dentigerous cysts are common noninflammatory developmental odontogenic cysts characterized by a radiolucent pericoronal area in conjunction with tooth eruption failure [37]. The most accepted theory of the etiology of a dentigerous cyst is fluid accumulation between the reduced enamel epithelium and the crown of the permanent tooth germ resulting from the pressure exerted by the erupting tooth on its own dental follicle [38]. Patients are mostly asymptomatic and therefore fail to detect the issue on their own. Cyst enlargement (>5 mm follicular-space diameter) [39,40] may cause swelling, mild sensitivity, tooth movement, and displacement [41]. To the best of our knowledge, there are fewer statistics on MM2-containing dental cysts, and more studies are related to impacted MM3 [42]. This study included 12 impacted MM2 associated with dentigerous cysts. Moreover, 91.7% of patients with cysts had Type III impactions, indicating that deeper impactions might be more susceptible to cysts.

Binary regression analysis showed that young developing MM2 and increased depth were risk factors for the presence of cysts around MM2 with eruption disturbances. Since the ORs gradually decreased with each developmental stage from Stages 8 to 10, which means the earlier the stage of MM2 development, the greater the chance of cyst formation. In other words, the incidence of cysts was lower in better-developed MM2, and the assessment of MM2 depth showed that deeper MM2 had a greater chance of cyst formation.

Miyawaki and Hyomoto et al. [43,44] showed that marsupialization for cyst could facilitate tooth eruption through rapid bone formation accompanied by pressure release and that eruption is related to root development, age, depth, and angle of impaction tooth.

The results of our study are applicable to all patients who have not erupted two years after the normal time of MM2 eruption or have only unilateral fully erupted MM2. This relatively rare study of MM2 eruption disturbances may provide dentists with an overall understanding to aid in early diagnosis and treatment planning.

## 5. Conclusions

1. The majority of MM2 with eruption disturbances were due to impaction, while the minority attributed to retention.
2. The majority of impacted MM2 with Type I or Type II were caused by insufficient space, while impacted MM2 with Type III were attributed to a variety of causes, including insufficient space, ectopic path, and cysts.
3. The majority of impacted MM2 in Type I and Type II were mesioangular, and Type III presented various angulations.
4. Cyst development was associated with greater impaction depth and earlier development stage of MM2. However, it was not associated with sex, age, presence of undercut, narrower posterior space, or angulation.

## Figures and Tables

**Table 1:** Patient demographics and baseline information based on impaction and retention.

Item/Eruption Disturbances	Retention	Impaction	Total	*p*-Value
Number of patients (*n*)	5	107	112	
Number of MM2 (*n*)	6	137	143	
Number of unilateral or bilateral (*n*)				>0.9999
Unilateral	4	77	81	
Bilateral	1	30	31	
Side (in unilateral patients) (*n*)				0.311
Left	1	45	46	
Right	3	32	35	
Sex (*n*)				0.081
Male	5	62	67	
Female	0	45	45	
Age (yrs)	21.50 ± 8.31	16.91 ± 5.95	17.45 ± 6.35	0.302

Age data were presented as mean ± SEM.

**Table 2:** Demographics and baseline information of the patients based on impaction and retention.

Item/Eruption Disturbances	Retention		Impaction			Total
	Primary Retention	Secondary Retention	Insufficient Space	Ectopic Eruption Path	Cyst of Jaw	
Number of patients (*n*)	2	3	87	9	11	112
Number of MM2 (*n*)	3	3	115	10	12	143
Number of unilateral or bilateral (*n*)						
Unilateral	1	3	59	8	10	81
Bilateral	1	0	28	1	1	31
Side (in unilateral patients) (*n*)						
Left	0	1	34	3	8	46
Right	1	2	25	5	2	35
Sex (*n*)						
Male	2	3	52	5	5	67
Female	0	0	35	4	6	45

**Table 3:** Measurements between types based on impaction.

Item/Impaction Type	Type I	Type II	Type III	Total	*p*-Value
Number of patients (*n*)	39	25	31	95	
Number of MM2 (*n*)	63	38	36	137	
Existence of MM3 (*n*)	56	34	33	123	
Number of unilateral or bilateral (*n*)					0.130
Unilateral	24	17	26	67	
Bilateral	15	8	5	28	
Side (in unilateral patients) (*n*)					0.701
Left	14	9	17	40	
Right	10	8	9	27	
Depth of mesial (mm, mean)	5.63 ± 3.20	8.26 ± 3.45	12.16 ± 4.20	8.05 ± 4.42	<0.001 **
Depth of distal (mm, mean)	0.55 ± 3.09	4.40 ± 2.78	9.70 ± 3.40	3.98 ± 4.84	<0.001 **
Distance from the MM1 distal to the ramus (mm, mean)	9.00 ± 3.47	7.80 ± 2.34	9.12 ± 3.05	8.38 ± 3.54	0.226
Risk factor (*n*)					<0.001 **
Insufficient space	60	37	18	115	
Ectopic eruption path	3	0	7	10	
Cysts of jaw	0	1	11	12	
Undercut (*n*)					<0.001 **
Undercut	62	36	20	118	
No-undercut	1	2	16	19	
Angulation (*n*)					<0.001 **
Horizontal	7	6	6	19	
Mesioangular	55	30	17	102	
Vertical	0	1	5	6	
Distoangular	1	1	5	7	
Other	0	0	3	3	
Haavikko Staging of MM2 (*n*)					0.061
R1/2	3	9	5	17	
R3/4	15	8	9	32	
Rc	24	7	7	38	
Ac	21	14	15	50	

** *p* < 0.01, depth data were presented as mean ± SEM.

**Table 4:** Binary regression analysis of cyst in impaction.

Independent Variable	Odds Ratio	*p*-Value	95% Confidence Interval	
Sex	3.76	0.136	0.66	21.38
Age	1.18	0.243	0.9	1.54
MM2 depth	51.92	0.000 **	7.26	371.38
Distance from the MM1 distal to the ramus	0.81	0.142	0.61	1.07
Undercut	0.98	0.984	0.21	4.68
Haavikko Staging (stage11)		0.043 *		
Stage8	229.97	0.005 **	5.30	9983.09
Stage9	113.47	0.010 *	3.09	4164.00
Stage10	42.30	0.030 *	1.42	1256.13
Angulation (Horizontal)		0.855		
Mesioangular	1.55	0.776	0.08	31.31
Vertical	5.66	0.406	0.10	337.60
Distoangular	6.98	0.357	0.11	436.76
Other	0.00	0.999	0.00	-

* *p* < 0.05, ** *p* < 0.01.

## Data Availability

The data presented in this study are available upon request from the corresponding author upon reasonable request.
